# Hepatoid adenocarcinoma of the stomach effectively treated with capecitabine with oxaliplatin as adjuvant chemotherapy: A case report and literature review

**DOI:** 10.1016/j.ijscr.2023.108963

**Published:** 2023-10-17

**Authors:** Jun Amioka, Senichiro Yanagawa, Yuji Yamamoto, Masahiro Nakahara, Shuji Yonehara, Toshio Noriyuki

**Affiliations:** a Department of Surgery, Onomichi General Hospital, 1-10-23 Hirahara, Onomichi, Hiroshima, Japan; b Department of Pathology, Onomichi General Hospital, 1-10-23 Hirahara, Onomichi, Hiroshima, Japan

**Keywords:** Hepatoid adenocarcinoma of the stomach, Chemotherapy, CapeOX, Surgery

## Abstract

**Introduction:**

Hepatoid adenocarcinoma of the stomach (HAS) is an alpha-fetoprotein (AFP)-producing gastric carcinoma (GC) with a hepatocellular carcinoma-like histology. HAS is a relatively rare type of GC, with liver metastases being more common than peritoneal dissemination in the recurrent form, and the poor prognosis.

**Presentation of case:**

We present the case of a 70-year-old patient who underwent distal gastrectomy for GC and immunohistologically diagnosed as HAS. The patient had an intravenous tumor thrombus at the proximal margin of the resected stomach. Owing to the low possibility of radical resection and high probability of liver metastatic recurrence, capecitabine with oxaliplatin (CapeOX) was started as adjuvant chemotherapy (AC). After three courses of CapeOX, oxaliplatin was discontinued due to adverse events (peripheral neuropathy, grade3) and capecitabine alone was continued for 3 years postoperatively. Six years after surgery, no local recurrence or distant metastasis was detected on imaging studies.

**Discussion:**

There is no established standard treatment for HAS. Recently, some studies have reported the efficacy of antimetabolites or platinum-based drugs as AC regimens. We thus decided to start a regimen consisting of a combination of antimetabolites and a platinum, i.e., CapeOX, which proved efficacious.

**Conclusion:**

CapeOX or capecitabine may be effective as AC for treating HAS.

## Highlights

• Hepatoid adenocarcinoma of the stomach (HAS) is rare type of gastric carcinoma (GC).
• There is no established standard treatment for HAS.
• Our patient, after distal gastrectomy, was started on capecitabine with oxaliplatin.
• Six years after surgery, no local recurrence or distant metastasis was detected.

## 1 Introduction

Hepatoid adenocarcinoma of the stomach (HAS) is a rare type of gastric carcinoma (GC), first defined by Ishikura in 1985 [1], with symptoms similar to those of normal GC (abdominal discomfort, fullness of anorexia, epigastric pain, vomiting, and weight loss).

HAS is an adenocarcinoma with a histologic structure similar to hepatocellular carcinoma.

In one report, hematoxylin and eosin (H.E) staining revealed an abundance of cells with eosinophilic cytoplasm and round nuclei, and immunostaining showed positive results for alpha-feto-protein (AFP), sal-like protein 4 (SALL4), and glypican-3 or hep-par 1(Hep1) [2].

Compared to conventional GC, liver metastatic recurrence is more common than peritoneal dissemination in patients with HAS, at rates of 46.3–75.6 % [[3], [4], [5]]. Owing to the infrequency of the disease and the difficulty in diagnosis, no standard treatment has been established, and there is no consensus on the chemotherapy regimen.

Here, we present a case of HAS with microscopical intravenous tumor thrombus for which capecitabine with oxaliplatin (CapeOX) was effective as adjuvant chemotherapy (AC).

This case report has been reported in line with the SCARE criteria [6].

## 2 Case presentation

**Fig. 1:**
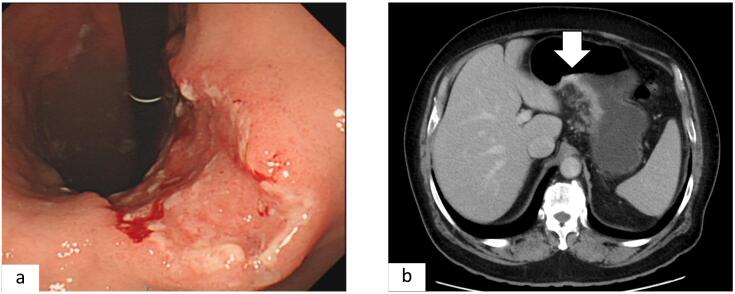
(a) EGD reexamination revealed a mass with a central ulceration at the lesser curvature extending from the gastric angle to antrum. (b) CT scans showed wall thickening with contrast effect on the anterior wall of the gastric lesser curvature and no clear distant metastases were observed.

A 70-year-old man with a history of hypertension and dyslipidemia visited our hospital for an ulcer lesion extending from the gastric angle to antrum on esophagogastroduodenoscopy (EGD). Repeat EGD revealed the presence of a tumor with a central ulceration at the lesser curvature extending from the gastric angle to the antrum (Fig. 1a) diagnosed as a poorly differentiated adenocarcinoma by biopsy at our hospital.

Blood examination data showed an elevated serum AFP level of 122.9 ng/mL (reference range: 0.89–8.78 ng/mL), while carbohydrate antigen 19–9 and carcinoembryonic antigen levels were within the reference levels. Computed tomography (CT) revealed wall thickening with contrast effect on the anterior wall of the gastric lesser curvature without invasion of other organs (Fig. 1b), with no distant metastases, and this was diagnosed as cT3N0M0 cStageIIB according to the Union for International Cancer Control (UICC) tumor node metastasis (TNM) classification 8th edition.

We performed laparoscopic distal gastrectomy with Roux-en-Y reconstruction and D2 lymph node dissection instead of total gastrectomy, because the distance from the esophagogastric junction to the oral margin of the tumor was approximately 5 cm.

**Fig. 2:**
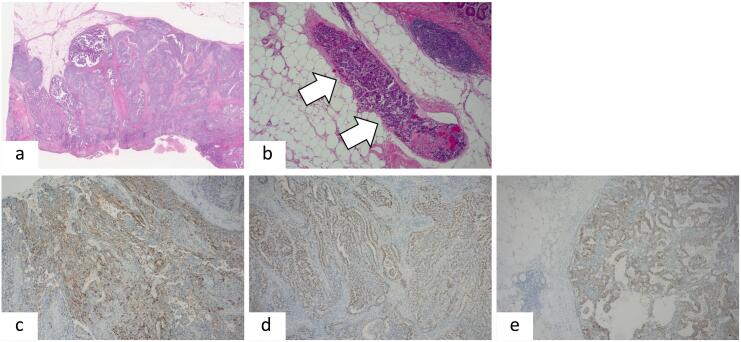
H.E staining showing polygonal tumor cells forming clear and abundant eosinophilic cytoplasm, separated by sinusoidal capillaries, and trabecular and intestinal-like structures (a) and there is intravenous tumor thrombus at the proximal resection (b). Immunohistochemical staining of tumor cells are broadly positive for AFP (c), SALL-4(d), glypican-3(e).

On H.E staining of the specimen obtained during gastrectomy showed polygonal tumor cells forming, clear and abundant eosinophilic cytoplasm, separated by sinusoidal capillaries and trabecular and intestinal-like structures (Fig. 2a). At the proximal margin of the resected stomach, an intravenous tumor thrombus was observed (Fig. 2b). Immunostaining revealed positivity for AFP (Fig. 2c), SALL4 (Fig. 2d), and glypican-3 (Fig. 2e). Based on these pathological findings, the tumor was diagnosed as HAS. Five lymph node (No.1, 3, 8a) metastases were also observed.

The patient was diagnosed with pathological stage IIIA (U,Less,TypeII,130 × 80 mm, hepatoid > pap, pT3, INFb, Ly1, V3, pPM1, pDM0[80 mm], pN2[5/42], CY0, P0) according to the UICC TNM classification 8th edition.

The patient was started on CapeOX as adjuvant chemotherapy. The CapeOX regimen consisted of oxaliplatin 130 mg/m^2^ on day 1 and capecitabine 2000 mg/m^2^ on days 1–14 with a rest period on days 15–21. After three courses of CapeOX, oxaliplatin was terminated due to peripheral neuropathy (grade 3) graded according to the Common Terminology Criteria for Adverse Events. Since there was a venous tumor thrombus with a high risk of liver metastasis recurrence, capecitabine was planned to be continued as long as possible considering its adverse effects. Capecitabine was terminated after 3 years, because the adverse effect of hand-foot syndrome, progressed and reached grade 2, interfering with the patient's daily life.

The patient was followed up on an outpatient basis with blood tests every 3 months, CT every 6 months, and EGD every year. Serum AFP was elevated preoperatively but fell to within the reference range and remained within the reference range thereafter. Serum CEA and CA19-9 remained within the reference range from the preoperative period. EGD showed no evidence of recurrence of HAS or development of new residual GC in the residual stomach and CT did not show any evidence of peritoneal dissemination, local recurrence, or distant metastasis for 6 years after surgery.

## 3 Discussion

HAS, a representative AFP-producing GC, which presents a hepatocellular carcinoma-like histology, was first defined by Ishikura in 1985 [1]. HAS is relatively rare, accounting for 0.55 % of all GC cases [7]. Serum AFP level can be used as an indicator of the effectiveness of postoperative treatment, in patients with HAS. Serum AFP is a useful tumor marker that correlates with prognosis [8].The histological features of HAS are similar to those of hepatocellular carcinoma, where the cells are enriched with eosinophilic cytoplasm and round nuclei on H.E staining and positive for AFP, SALL4, glypican-3 on immunohistochemical staining. Clinically, the most common site of metastasis is the liver, with a reported rate of 46.3–75.6 % [[3], [4], [5]]. The 3-year survival rate is reported to be 7.36 % [9]. Regarding intravenous thrombus, Yang et al. reported that intraportal tumor plugs were noted on CT in 7 (31.8 %) of 22 patients with HAS, of whom 5 of 7 had intravenous tumor thrombus and liver metastases, indicating that intravenous tumor thrombus is associated with a high rate of liver metastases [10].

Owing to the rarity and paucity of data in the literature, no effective treatment has been established for HAS. However, R0 resection and chemotherapy have been reported to affect prognosis [7]. As shown in Table 1, some patients achieved good prognosis with R0 resection including hepatectomy [9,[11], [12], [13], [14], [15], [16], [17]]. Several studies have used platinum-based chemotherapy [9,11,12,15].

**Table 1:** Review of cases of HAS with R0 resection and chemotherapy.

Case	Source [Refs] Year	Age Gender	Surgical procedure	Immunohistochemically positive	Liver metastasis	Chemotherapy regimen	Outcome (period after surgery)
1	Nuevo et al. [9] 2012	67 Female	Radical resection	AFP	No	CDDP/Capecitabine/EPI	Alive (12 months)
2	Ahn et al. [11] 2013	68 Male	Radical resection	AFP	No	S1, CDDP/Capecitabine, FOLFILI	Alive (21 months)
3	Mahajan et al. [12] 2014	60 Male	Radical resection	AFP	No	CDDP/5-FU	Alive (12 months)
4	Liu et al. [13] 2015	47 Male	Radical resection	AFP	No	None	Alive (12 months)
5	Zhou et al. [14] 2015	72 Male	Radical resection	Hep1	No	Capecitabine/fluorouracil	Alive (unknown)
6	Shen et al. [15] 2016	70 Male	Radical resection	AFP	Yes	Capecitabine/oxaliplatin	Alive (unknown)
7	Yoshizawa et al. [16] 2017	61 Male	Radical resection	AFP, SALL4	Yes	S1	Alive (unknown)
8	Arakawa et al. [17] 2017	56 Male	Radical resection	AFP	No	S1, Paclitaxel, Irinotecan, Ramucirumab	Alive (19 months)

EPI: Epirubicin Hydrochloride CDDP: cisplatin FOLFILI: fluorouracil, folinic acid, irinotecan 5-FU: 5-Fluorouracil.

In our patient, the distance from the oral margin of the tumor to the resected gastric margin was 2 cm and, pathologically, the mucosa was normal, but, tumor thrombus was found at the proximal margin of the resected stomach; hence, there was a high possibility that R0 resection had not been performed, so we considered performing total residual gastrectomy with curative intent. However, the probability of liver metastasis was considered high because of the serum AFP level of ≧40 as reported by Qu et al. [8], and the high rate of liver metastatic recurrence of HAS associated with intravenous tumor thrombus as reported by Yang et al. [10], we decided to start chemotherapy rather than performing total resection of the residual stomach. Based on previous literature [9,[11], [12], [13], [14], [15], [16], [17]] and the results of J-CLASSIC-PII trial [18], we decided to introduce a regimen consisting of a combination of antimetabolites and platinum agent, and selected CapeOX. After three courses of CapeOX, capecitabine was administered for 3 years, depending on the adverse effects, because four of the eight references used capecitabine and continued capecitabine monotherapy [9,11,14,15]. After completing the course of capecitabine, the patient was followed on an outpatient basis. The patient survived for 6 years postoperatively without local recurrence or distant metastasis.

## 4 Conclusion

The patient had an intravenous tumor thrombus and a high risk of recurrence of liver metastasis. However, the patient did not show any signs of recurrence. Our case report suggests that the CapeOX or capecitabine regimen may be an effective option for AC for HAS.

## Consent

Written informed consent was obtained from the patient for publication of this case report and accompanying images. A copy of the written consent is available for review by the Editor-in-Chief of this journal on request.

## Provenance and peer review

Not commissioned, externally peer reviewed.

## Ethical approval

This study protocol was reviewed and the need for approval was waived by the Institutional Review Board of the Onomichi General Hospital Ethics Review Committee.

## Funding

This research did not receive any specific grant from funding agencies in the public, commercial, or not for profit sectors.

## Author contribution

**Jun Amioka**: Conceptualization, Methodology, Software, Validation, Formal analysis, Investigation, Resources, Data Curation, Writing-Original Draft, Writing-Review & Editing, Visualization.

**Senichiro Yanagawa**: Conceptualization, Methodology, Validation, Data Curation, Writing-Original Draft, Writing-Review & Editing, Supervision, Project administration. **Yuji Yamamoto**: Validation, Writing-Review & Editing.

**Masahiro Nakahara**: Validation.

**Shuji Yonehara**: Validation, Date Curation, Writing-Review & Editing.

**Toshio Noriyuki**: Project administration.

## Guarantor

Senichiro Yanagawa.

## Research Registration Number

Not applicable.

## Conflict of interest statement

The authors have no conflicts of interest to declare.

## Data Availability

All data generated or analyzed during this study are included in this article. Further enquiries can be directed to the corresponding author.
